# Interrupted breeding in a songbird migrant triggers development of nocturnal locomotor activity

**DOI:** 10.1038/s41598-018-23834-0

**Published:** 2018-04-03

**Authors:** Andrey Mukhin, Dmitry Kobylkov, Dmitry Kishkinev, Vitaly Grinkevich

**Affiliations:** 1 Biological Station Rybachy of Zoological Institute RAS, 238535 Rybachy, Kaliningrad reg., Russia; 2 Institut für Biologie und Umweltwissenschaften, AG Neurosensorik/Animal Navigation, University of Oldenburg, Oldenburg, 26111 Germany; 3 Department Molecular Physiology and Biophysics, Immanuel Kant Baltic Federal University, Kaliningrad, Russia; 4 School of Biological Sciences, Bangor University, Bangor, Gwynedd, LL57 2UW UK

## Abstract

Long-distance avian migrants, e.g. Eurasian reed warblers (*Acrocephalus scirpaceus*), can precisely schedule events of their annual cycle. However, the proximate mechanisms controlling annual cycle and their interplay with environmental factors are poorly understood. We artificially interrupted breeding in reed warblers by bringing them into captivity and recording birds’ locomotor activity for 5–7 days. Over this time, most of the captive birds gradually developed nocturnal locomotor activity not observed in breeding birds. When the birds were later released and radio-tracked, the individuals with highly developed caged activity performed nocturnal flights. We also found that reed warblers kept indoors without access to local cues developed a higher level of nocturnal activity compared to the birds kept outdoors with an access to the familiar environment. Also, birds translocated from a distant site (21 km) had a higher motivation to fly at night-time after release compared to the birds captured within 1 km of a study site. Our study suggests that an interrupted breeding triggers development of nocturnal locomotor activity in cages, and the level of activity is correlated with motivation to perform nocturnal flights in the wild, which can be restrained by familiar environment.

## Introduction

On migration, many songbird species profoundly change their diel rhythm under the control of their intrinsic circannual programs and perform nocturnal long-range flights^1^. This adaptive change of a daily activity rhythm several times per year allows migratory birds to use seasonally available resources in a breeding area in the daytime, whereas a nocturnal flight enables them to move faster and with less predation risks along a migratory route, using the daytime for foraging^2,3^. Change to the so-called nocturnality (a behavior characterized by the presence of locomotor activity both day and night) is believed to be governed by endogenous programs^4–7^. Based on the results of cage experiments with animals isolated from external stimuli and kept in a constant photoperiod, some authors suggested that nocturnality is only observed during migratory seasons^8^. As first assumed^9–11^ and then shown experimentally^6^, the manifestation of migratory restlessness seems to be based on interaction of two separated biological oscillators^12,13^, but see^14^. Bartell and Gwinner^6^ showed that captive garden warblers demonstrating strictly diurnal activity controlled by endogenous programs in a certain time of their annual cycle gradually shift a part of their locomotor activity to the nighttime. This shift reflects a step-by-step increase in phase angle between the two previously coupled oscillators controlling diurnal and nocturnal activity. According to the hypothesis of Bartell and Gwinner^6^, nocturnality does not appear over one night but rather is gradually developing several days, and so is nocturnal locomotor activity.

Previous studies, however, have not addressed the question whether the gradual shift of locomotor activity of captive birds from strictly diurnal to both diurnal and nocturnal activity is restricted to migratory seasons or this change in activity could be triggered at any step of the bird’s annual cycle. In our prior study^15^, we radio-tracked free-living Eurasian reed warblers, a common European long-distance songbird migrant, and either simulated a nest loss by removing their nestlings to captivity or translocated breeding birds for up to 21 km from their nests. All birds were radio-tagged and their day/night movements in the field were tracked to reveal changes of their activity rhythm and observe homing movements. Most of the birds with interrupted breeding performed night-time flights either back to their nests (translocated individuals) or probably to search for new places to breed (birds with artificial nest loss who most probably started searching for alternative nest site). However none of the birds did night flights during the first two nights upon displacement from a nest or artificial nest predation. The night-time departures were recorded from the third night onward^15^. Based on that study, we hypothesized that changes of nocturnal migrants’ activity schedules are far more flexible than previously assumed and can be observed besides migratory seasons.

In the present study, we had two objectives: (1) to test whether birds with artificially interrupted breeding and a high motivation to return to their nests (taken into captivity at the breeding stage with 4–6 day nestlings) are gradually developing locomotor activity at the night-time similar to a development of migratory restlessness of caged birds during a migratory season. The time needed for the gradual development of nocturnal activity could be exactly responsible for the time lag before nocturnal departure observed in the radio-tagged free-living birds in our previous study^15^; (2) to test the hypothesis that environmental factors associated with breeding territories can modify the development of nocturnal activity in captivity and affect motivation to perform nocturnal flights in freely moving reed warblers. Some previous laboratory-based studies have suggested that nocturnal migratory restlessness could be governed not only by endogenous but also external factors (food availability^16–18^; mate presence^19,20^).

To achieve these two goals, we captured breeding reed warblers during their nesting near our study site or at a distance of 21 km from it, placed birds into captivity with or without access to the familiar breeding territories for 5–7 days until well developed cage nocturnal activity. Then we released and radio-tracked them in the wild. We analyzed the development of nocturnal locomotor activity of captive birds and the flights of released birds with respect to whether they were able to perceive environmental factors associated with familiar breeding territories or not.

## Material and Methods

### Ethical statement

All experiments were approved and conducted according to the relevant legislation and guidelines. The experiments complied with the current laws of the Russian Federation and institutional guidelines of Zoological Institute of the Russian Academy of Sciences and its Statute (approval letter No 88, 07.04.2007). All experimental procedures were according to All Union State standard (ГОСТ № Р53434-2009 “Principles of good laboratory practice”) of Russian Federation. All culling of experimental birds were permitted by Forest and Nature Protection Agency of Kaliningrad Region, Russia (№ 3-03-2010).

### Study site and experimental birds

The study was carried out at the Biological Station Rybachy on the Courish Spit of Baltic Sea, Russia (55°00-09′N, 20°34-51′E) in 2009–2012. Because we expected a different nocturnal activity depending on familiar and unfamiliar surroundings, two groups of birds were tested in two experiments: local and translocated ones. The local birds were caught in the reed beds near Rybachy village, 0.03–0.25 km from the village, the translocated ones were caught 21.2–21.3 km away from the housing place. Only males were used in this study to reduce the risk of starving for nestlings (females tend to be more motivated to feed nestlings). The birds were caught by an automatic clapping trap at their nests when their nestlings were 4–6 day old because at this stage adult males are most motivated for homing (returning to their nests). All experimental birds received food (mealworms) and water *ad libitum*. See information about cages and captivity conditions below.

### Birds kept indoors before release

8 local and 13 translocated birds were housed indoors in a room without windows (no viewing of the surrounding area) in individual non-magnetic plastic cages 61 × 40 × 40 cm (Joko-Systemtechnik GmbH, Germany) under artificial light conditions simulating the local photoperiod (day light: >500 lux, night light: <<0.2 lux with 40 min “twilight” transition). Both the local and translocated birds were kept in the cages 5–7 days until their nocturnal activity was well developed. During the captivity time, their locomotor activity was recorded (see “Recording locomotor activity” section below for more details). As we could not release birds at night without significant handling stress (birds would have been caught in indoor cages and then brought outside) and the stress could affected night take-off behavior, birds equipped with radio-transmitters were released at the study site early in the morning (outdoor birds could be released with less stress and were released at night-time, see below). After release their movements were radio-tracked (see more details in “Radio-tracking” section below).

### Birds kept outdoors before release

13 local and 11 translocated birds were radio-tagged and housed outdoors in individual non-magnetic wooden cages until their nocturnal activity was well developed (5–7 days). The outdoor cages of 57 × 40 × 40 cm dimensions were covered by synthetic net and fixed on a wooden 2.5 m high platform with access to visual, acoustic, geomagnetic cues and a view onto local birds breeding habitat. During the captivity time birds’ locomotor activity was recorded by infra-red sensors (see “Recording of locomotor activity” section below). When both the local and the translocated birds showed a well-developed nocturnal activity they were released at night-time by a remotely opening cage lid (to reduce stress due to handling effect). Such a mild stress of release at the night-time was possible only for the outdoor birds and not for the indoor birds (see above). All releases were performed under favorable weather conditions: clear sky, wind velocity <<3 m/s. The birds’ movements were then radio-tracked.

### Recording of locomotor activity

To record locomotor activity, both indoor and outdoor cages were equipped with passive infra-red sensors (S-230A Conrad Electronic GmbH, Germany). These sensors do not notice activity of a bird sitting at the same place but do notice moving of a body centre (e.g. jump from perch to perch or take-off attempts). The cages were additionally supplied with a video recording system in 2010–2012. The night-time locomotor activity was quantified as a number of active intervals (1 min bin) between midnight and the end of 40 min morning twilight transition (indoor birds) or the end of morning civil twilight (outdoor birds). Midnight was taken as an arbitrarily starting time point (the choice of a time reference point is not crucial here) and due to the easiness to quantify birds’ locomotor activity because it started developing from the late night hours and was gradually shifting towards midnight. 1 min interval was determined as an active one if a bird had one or more hops over a given minute. For the outdoors birds, the last night was excluded from the analysis because it was not a full night for the birds (they were released between 1:00 and 2:30 am).

### Radio-tracking

All released birds were equipped with ~0.7 g (<5% of their body weight, which is the standard for telemetry studies^21^) radio-transmitters (LB-2 Holohil Syst. Inc., Canada) using a leg-loop harness^22^. We used beeper radio-transmitters (each bird had individual radio frequency). We used 3-element directional Yagi antennas and both handheld and automatic radio receivers (handheld: R2000 Advance Telemetry Systems, TRX2000S WildLife Inc. USA; automatic: R4500 Advance Telemetry Systems and model 10–200 Sparrow Systems, USA). When releasing at night birds’ movements were detected from a nearby 15 m tower by manually detecting a bearing of Yagi antenna with the strongest signal. We considered that a bird had departed when no signal was recorded after a take-off during the night of release and birds were not detected by ground search within 5 km around release site. Once bird departed, its presence/absence nearby its nest was checked next morning. The presence of the released birds during the day-time at the releasing site was controlled by automatic radio receivers attached at the 15 m tower dominating over the release site and permanently scanning for the radio signals during the lifespan of the radio-tags.

### Statistics

All statistical calculations were performed in R^23^ using packages “nlme” and “MASS”. To analyze a development of nocturnal activity (coded as “Shift” in our models) we used linear mixed effects model (LME) according to the described protocol^24^, since this method deals with a longitudinal data and implements a repeated measurements’ design. “Day” (day of experiment, 1–6), “Place” (Outdoor/Indoor) and “Condition” (Local/Translocated) were used as explanatory variables in a fixed part of the model. Effects of “Day” and “ID” (individual) were included in the random part of the model. Optimal random structure (random intercept and slope model) was selected according to the lowest Akaike Information Criterion (AIC) of the corresponding model (2165.3 vs. 2204.3 respectively). Since heteroscedasticity in residuals between groups was detected, we modified our model and added a weight parameter for variances in all “Day” * “Place” combinations.

We progressively reduced the “beyond optimal” model with all possible interactions between factors by using maximum likelihood estimation method and backward model selection (selecting models with the lowest AIC) until the most parsimonious model was estimated. The parameters of this final optimal model were estimated using restricted maximum likelihood method. We applied F-statistics of ANOVA to obtain significance levels for each factor with a p-level <0.05 considered to be statistically significant. As “anova” function applies sequential testing, significance level can depend on the order of factors within the model. In our optimal model, a sequence of entrance did not affect the results. To describe the day-by-day difference in nocturnal activity development between groups, we used a nonparametric Wilcoxon Signed Rank Test for repeated measurements and compared the first day in captivity with each subsequent day. To compare how many local and translocated birds took off after release we used Yates-corrected χ^2^ test. The datasets analyzed during the current study are available from the corresponding author on request.

## Results

Once captured at their nests and transferred to captivity, all the birds, both indoors and outdoors, started gradually, over a few days (Figs S1–S4), developing nocturnal locomotor activity. Our linear mixed-model analysis revealed significant effect of all fixed factors (“Day”, “Place”, “Condition”, Tables 1 and 2) meaning that activity was shifting day-by-day and general amount of activity was different for indoor/outdoor and local/translocated groups. The ad hoc analysis of video recordings from caged birds revealed several types of locomotion during the nocturnal activity (e.g., movement along a perch, wing whirring, head scans, fluttering and attempts to take-off). We did not however quantify these locomotor acts throughout all the nights. Except of the movements along a perch, all these types of behavioural acts were absent during the day when the prevailing type of locomotion was perch hopping.

**Table 1:** Result of the backward selection of the fixed part of the Linear mixed-model. Models are sorted by Akaiki Information Criterion (AIC) with the best model with the lowest AIC placed below. Here is the development of nocturnal activity (“Shift”) depends on day of captivity (“Day”), place where birds were housed (“Place”: Outdoor/Indoor), surrounded conditions (“Condition” Local/Translocated) and interactions of those factors.

Model (Shift ~ …)	AIC
~Day + Place + Condition + Day:Place + Day:Condition + Place:Condition + Day:Place:Condition	2165.3
~Day + Place + Condition + Day:Place + Day:Condition + Place:Condition	2163.6
~Day + Place + Condition + Day:Place + Place:Condition	2161.7
~Day + Place + Condition + Day:Place	2161.0

**Table 2:** F-statistic (ANOVA) of the most parsimonious model (Shift ~ Day + Place + Condition + Day:Place) using restricted maximum likelihood estimation method.

Factor	F-value	*P*-value
Day	68.134	<0.0001
Place	7.578	0.0089
Condition	6.608	0.0141
Day:Place	23.441	<0.0001

**Figure 1:**
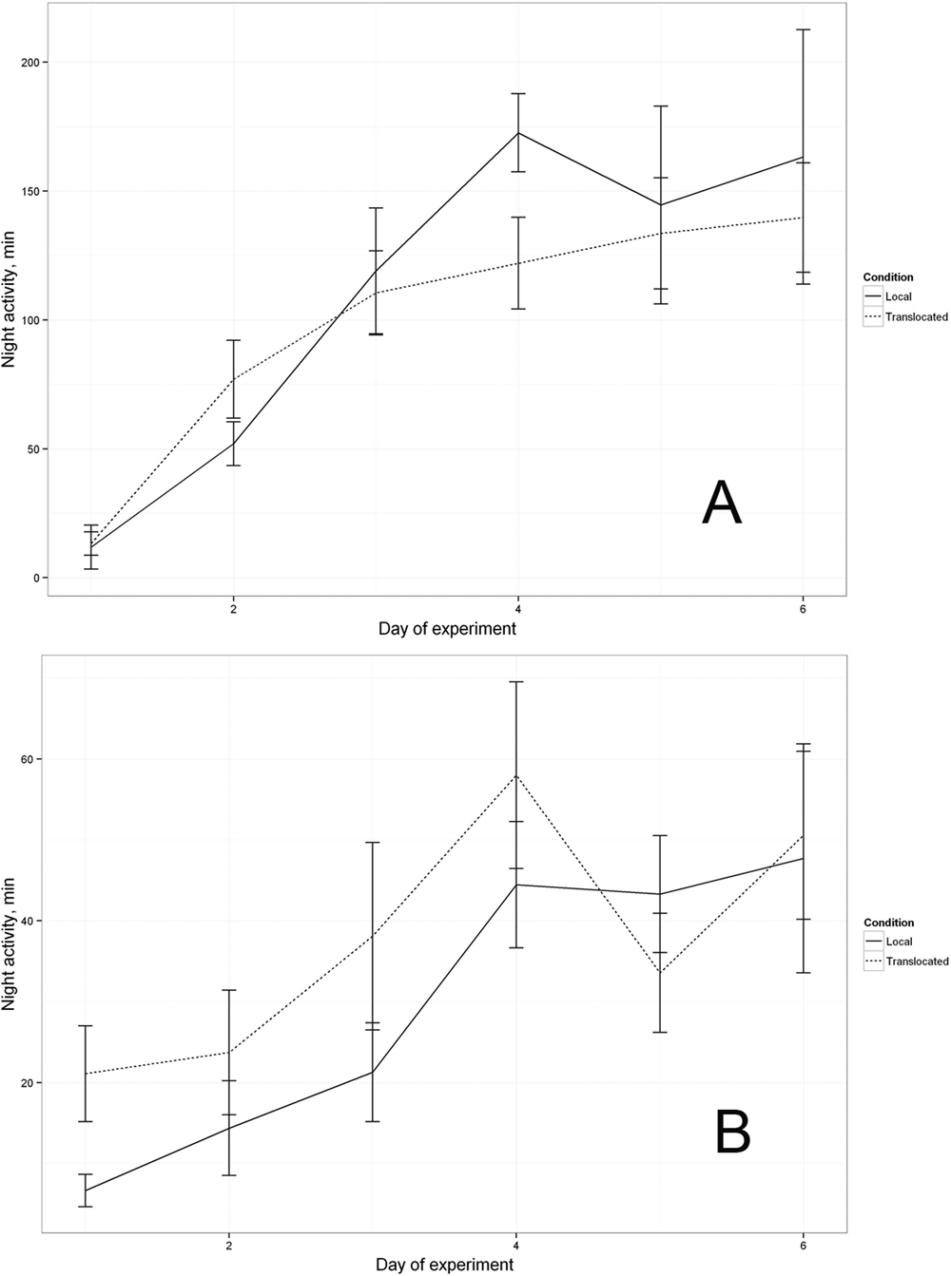
Development of nocturnal activity in local (solid line) vs. translocated (dotted line) birds for indoor (**A**) and outdoor (**B**) housing. The line represents a mean active period for all birds (in minutes per night) with error bars (SEM).

Both the local and translocated birds kept indoors were not only more active during the night-time compared to the local and translocated birds kept outdoors, but also developed nocturnal activity faster (significant effect of the factor “Day:Place”, Figs 1A and 3). Nocturnal activity of the indoor birds became evident already on the second day (Wilcoxon Signed Rank Test, *P* = 1.66E-05) and was gradually increasing up to the fourth day (on average over 100 active minutes per night, Fig. 1A (see Supplementary Fig. S1 as an example for the local birds’ group and Fig. S2 as an example for the translocated birds’ group)).

**Figure 2:**
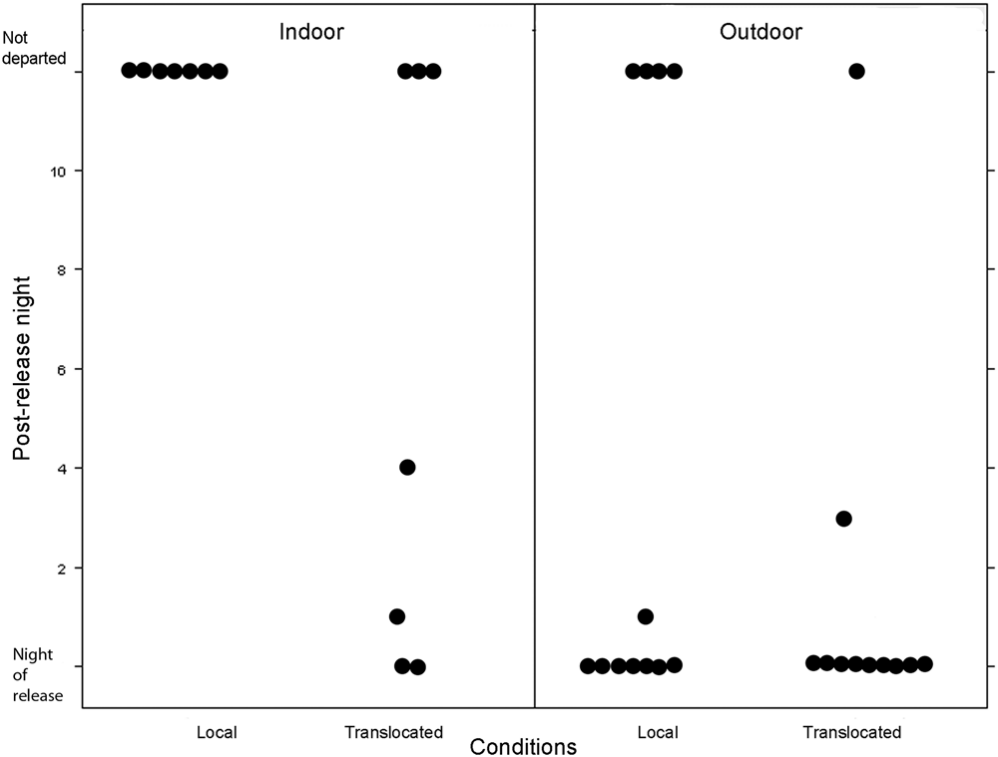
Distribution of nocturnal departures. 0 – night of release; 12 – non-departed birds (indoor – left panel, outdoor – right panel).

Once the indoor captive birds had developed nocturnal activity (Figs S1, S2), 7 local and 9 translocated birds were released with radio-transmitters. All the indoor kept birds were released early in the morning and their movements at the release site were radio-tracked. From the local birds previously kept indoors, none departed from the study site by a nocturnal flight after release (Left panel in Fig. 2) and just one bird engaged in nocturnal flights. The latter bird was active only during the first night after release, but then ceased nocturnal activity. However, a few radio signal changes without real flights were recorded by this bird during the 1^st^ and 2^nd^ post-release nights suggesting that this bird was awake. From the 9 translocated birds previously kept indoors, 2 lost their radio-transmitters shortly after release, 2 departed from the study site at the first night after release, 1 bird departed on the 2^nd^ night after release (after an aborted attempt to fly during the first night), 1 bird left during the 4^th^ post-release night, and 3 did not leave until the end of transmitter lifespan. For the latter ones, weak radio signal changes were recorded during 2–3 nights after release without actual flights (Left panel on Fig. 2).

**Figure 3:**
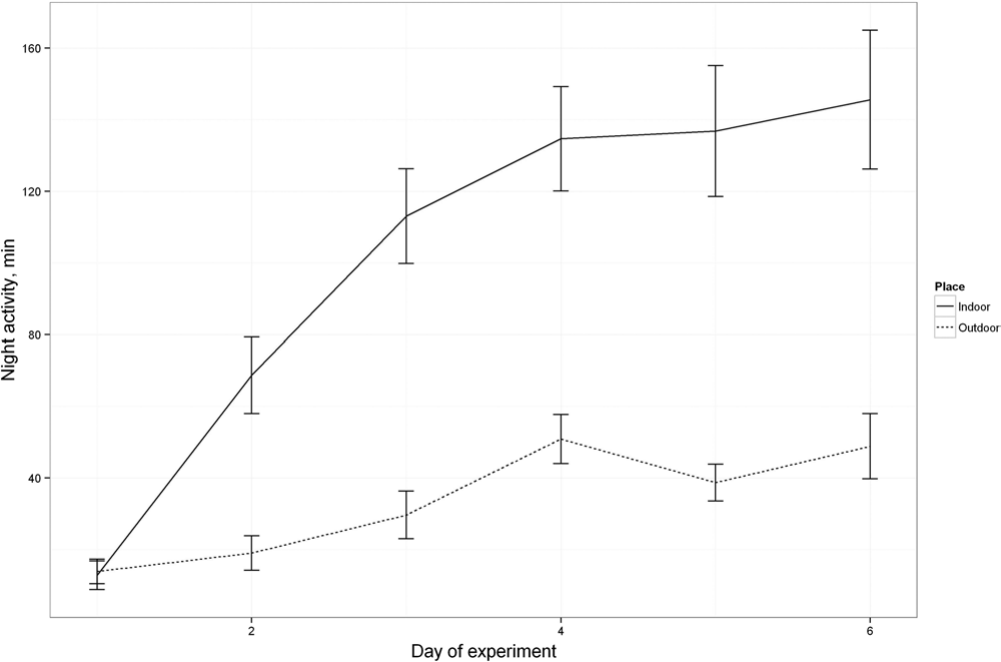
Development of nocturnal activity in groups, placed indoors and outdoors. The line represents a mean active period for all birds (in minutes per night) with error bars (SEM).

The birds kept outdoors developed their nocturnal activity in cages as well. However they were developing it slower than the indoor birds (Figs S3, S4 vs S1, S2). It became significant only on the fourth night of experiment (local: *P* = 0.004, translocated: *P* = 0.039 Fig. 1) and the level of night-time locomotor activity was overall lower than by the indoor birds (Fig. 3). Although the factor “Condition” (local vs. translocated) does have a significant effect in the optimal model (Tables 1 and 2), this effect is obvious only in the outdoor birds where the translocated birds showed more activity than the local ones (Fig. 1B). Once the 21 outdoor birds (13 local and 11 translocated) developed nocturnal activity in captivity (see Supplementary Figs S3 and S4 as example of nocturnality development in local and translocated groups, respectively), they were released at night and their take-off behaviour was radio-tracked at the release site and within 3 km during the following days. From the 13 local birds, 8 departed from the study site after release (7 during the night of release and one on the 1^st^ post-release nights) and 4 were not engaged in nocturnal flights at all up to the end of transmitter lifespan. From the 4 not departed birds, one took-off immediately after release, but 1.5 hours later it returned to the study site and showed no nocturnal activity ever since (see right panel on Fig. 2). From the 11 translocated and kept outdoors birds, 9 departed during the night of release, 1 individual departed during 3^rd^ post-release nights, and 1 bird, which had shown no developed nocturnal activity in captivity before release, was staying at the study site until its radio-tag failed (see right panel on Fig. 2) and showed no attempts to take off at the night-time. Five of the translocated birds were confirmed near their nests during the following day by radio-tracking.

Taken together, most of the translocated birds developed nocturnal locomotor activity in cages took off during the night of release (Table 3). Contrary to that, the local birds kept indoors prior release did not perform nocturnal flights at all whereas a good proportion of those kept outdoors (8 out of 13) departed during a release night or at the 1^st^ post-release night. The translocated birds departed more often after release compared to the local birds (Yates’ chi-squared test = 4.105, p = 0.04276), and the birds kept in the outdoor cages departed more often than the birds released from indoor cages (χ^2^ test with Yates correction = 6.03, p = 0.014).

**Table 3:** Number of birds demonstrated take-off behaviour.

		Departure	
		No	Yes
Indoor	Local	7	0
	Translocated	3	4
Outdoor	Local	5	8
	Translocated	1	10

## Discussion

Eurasian reed warblers are nocturnal, solitary migrating species and their migratory nocturnality is most probably governed by endogenous timing program as in other migratory songbird species studied so far^8^. Four times per annum they change their circadian rhythm of activity to be nocturnal during migration and diurnal during breeding season. Recently it was discovered that reed warblers’ night life is more extensive: they perform nocturnal flights in response to environmental factors like disturbance during breeding^15^ and explore the vicinity of their home ranges during pre-dawn hours before migration^25^. However, the proximate mechanism of this “out of migratory season” nocturnality is unknown. In our study, we first aimed to observe how nocturnal locomotor activity develops in caged birds that were captured while breeding and highly motivated to return to their nests. Our present data clearly show that reed warblers with artificially interrupted breeding and kept in captivity for 5–7 days were gradually developing locomotor activity at the night-time. In our experiment, nocturnal locomotor activity in caged reed warblers first appeared during pre-dawn hours and then was drifting towards midnight (see Figs S1–S4 as examples). Noteworthy, a similar pattern of locomotor activity development has been previously reported for an emerging migratory restlessness recorded in other captive songbird migrants^6,8,26^.

Why reed warblers need to develop nocturnal activity to perform short-distance (up to 21 km in our case) flights remains an open question, and the mechanism and the function of nocturnality during breeding period are unknown (e.g. Mukhin *et al*.^15^ for in depth discussion). Below we discuss two hypotheses but this list of not exhaustive. One hypothesis is inspired by the studies with captive songbird migrants during non-migratory seasons (e.g. wintering), and it assumes that the nocturnality outside migratory seasons is a general facultative response to stress^27,28^, food change and/or food deprivation^16^ or the other stressogenic factors that would force birds to leave a particular site at the night-time (the stress hypothesis). In our study, the birds were supplied with mealworms *ad libitum* and started eating during the first day in captivity. Therefore, we suggest that a stress due to food deprivation is unlikely to be the case but because we did not monitor the stress level (e.g. by corticosterone), and the birds in our study were prevented from seeing their mating partner, nestlings and the habitat, therefore we cannot rule out the stress hypothesis. Alternatively, one may suggest that there is a common mechanism for both migratory and breeding time nocturnality. We would like to draw attention to a couple of similarities in the development of nocturnal activities on migration and after an artificially disrupted breeding: 1) in both cases, the night-time locomotor activity starts gradually emerging from the late night hours and shifting towards midnight (Figs S1–S4) and 2) both migratory and breeding time nocturnality are manifested in similar stereotypical body movements (e.g., fluttering, whirring, head scans). These similarities may suggest that both the migratory and the summertime time nocturnal activity is governed by the common underlying mechanism but the difference between those two nocturnal activities is that the summertime nocturnal activity unpredictably emerges once triggered by external factors like aborted breeding whereas migratory restlessness is scheduled and ruled by changes in life-history stages. For this ‘common mechanism’ hypothesis, one may suggest that reed warblers (and maybe other nocturnal bird migrants) prefer to solve spatial tasks (e.g., returning to a nest, searching for a new nesting site or performing a migratory flight) at night and that both the summertime nocturnal activity and migratory restlessness may have the same endogenous (changes in biological oscillators) control but different exogenous modifying factors (cues associated with breeding for the former and photoperiod and/or light intensity for the latter). We would like to stress out that the present study did not aim to directly challenge either of the above mentioned hypotheses and that will be in focus of the future studies.

Our second goal for this study was to test the hypothesis that some environmental factors associated with breeding territories can modify the development of nighttime locomotor activity and affect motivation to perform nocturnal flights in freely moving reed warblers. It is worth noting that all but one of the birds, irrespective of the captivity type (outdoors or indoors) developed nocturnal activity in cages. Even reed warblers caught in Rybachy village and kept outdoors in the immediate vicinity of their nests did become restless at night after four days in captivity. It supports the idea that a loss of a nest and/or a mating partner is the main and overriding factor responsible for the changes to nocturnality during the breeding season. At the same time, our results strongly suggest that factors associated with a breeding site heavily affect the development of nocturnal activity in captivity because the indoor birds without access to the view, acoustic stimuli and odours of their breeding habitat developed nocturnality earlier than the outdoor birds and were more active in cages. Difference in the stress level might be an alternative or additional explanation but the stress level has not been measured in the present study and should be studied in the future.

As all experiments were performed during breeding period, taking the birds into captivity means an interruption of breeding. Results of this study together with our previous work^15^ allow us to conclude that removal from breeding is exactly the trigger which starts the development of nocturnality. What specific cue associated with the interrupted breeding governs this development? We suggest that the absence of a mating partner, nestlings, and a view of a nest or the combination of all these factors (the factors associated with housing in captivity) could initiate it. As our post-release radio-tracking observations showed, the level of cage locomotor activity was indeed correlated with the number of attempts to fly at night: birds’ with more cage activity were more likely to depart by a night-time flight from the release site.

Interestingly, the spatial behaviour of birds with developed nocturnal activity after release to nature was correlated with the possibility to immediately return to a nest. The local birds, especially from the indoor group (see the discussion on the local birds kept outdoors below), tended not to depart from the study site whereas almost all the translocated birds with their nests located at the distance of 21 km left the release site during the first 2 nights, and 5 of them were confirmed near their nests during the following day by radio-tracking. That means that they were heading towards their home territories soon after the nocturnal departure. Why the possibility to return to a nest immediately did abort nocturnal departure flights in the local birds but did not in the translocated ones? Previous studies with captive songbirds have suggested that the beginning of reproductive activity plays the major role in terminating nocturnal restlessness at the end of a spring migration^20^, and the main factor of it is the presence of a mating partner^19^. Based on that, we hypothesize that the social contacts with a mating partner and/or nestlings presented in nearby reed beds immediately reduced the motivation to depart and perform nocturnal flights among the local birds but the translocated birds lacked this factor.

The most striking difference in take-off behaviour after release was between the local birds kept indoors and the local birds kept outdoors. While in the indoor group all local birds ceased nocturnality after release early in the morning, 8 out of 13 birds from the outdoor group performed nocturnal flights when released at night. Such a difference within the local birds could be explained by the time of release. The local reed warblers kept indoors were released during early morning hours (see Methods for the rationale). During that time it was already too late for them to take-off (average time for night departures of reed warblers is 3 hours 38 min after sunset^15^). Release site was only 30–250 m away from their nests, so during the day-time they could easily find their mating partners and broods. Returning to normal breeding activity could block nocturnality in these local birds as it does during terminal part of spring nocturnal activity^20^. On the contrary, a large part of the local birds kept outdoors and released at the night-time performed nocturnal flights and departed the study site right away. One may speculate that without the possibility to estimate the presence/absence of their nest sites at night^29^ these birds with well-developed nocturnal activity had a strong motivation to depart immediately after release. Evidence of this could be a short-term departure of a local bird, which then returned after 1.5 hour absence and showed no attempt to leave the site afterwards. This bird might immediately unify with the mating partner and brood and lost motivation to depart.

To sum up, our data suggest that the summertime nocturnal activity in previously free-living but then caged European reed warblers induced by interrupted breeding develops gradually starting from the late hours of the night shifting towards the mid-night and associated with such locomotor acts as whirring, fluttering, head scans (also reported for migratory night-time activity^30–33^. In the past, some authors have argued that the induced nocturnal activity is a caged representation of a real nocturnal flight (see also Eikenaar *et al*.^34^; Schmaljohann *et al*.^35^). Our data show a clear correlation of the level of caged locomotor activity and post-release take-offs that corroborates this hypothesis. Due to the fact that at least some of the translocated birds departed from the release site were later found at their nests, this summertime nocturnal activity in reed warblers seems to be used for solving spatial and/or navigational tasks (e.g., finding a nest after translocation). Noteworthy, that Bäckman *et al*.^36^ reported similar midsummer night flights in one Red-backed shrike as well as Roth *et al*.^37^ reported the similar behavior for a few female nightingales what may indicate that such summertime nocturnality is rather common phenomenon among songbirds. In the future, we plan to use the induced nocturnal activity to study the role of natural factors used for short-distance navigation in the summertime. Last but not least, despite the fact that captivity always triggered the nocturnal activity in breeding reed warbler males, we also found that some external factors associated with a breeding site (e.g., local view, sounds, odours and re-union with a mating partner and/or nestlings) can modify the development of this nocturnal activity and even completely block it. The relative weights of these factors should be in the focus of further studies.

## Electronic supplementary material

Supplementary figures
