# Supplementary material for: Interrupted breeding in a songbird migrant triggers development of nocturnal locomotor activity
Source: Sci Rep. 2018 Apr 3;8:5520. doi: 10.1038/s41598-018-23834-0 (PMC5882773; doi:10.1038/s41598-018-23834-0)
Supplement: Supplementary file 1 — Supplementary figures [file 41598_2018_23834_MOESM1_ESM.pdf]

Interrupted breeding in a songbird migrant triggers development of nocturnal locomotor activity

Andrey Mukhin, Dmitry Kobylkov, Dmitry Kishkinev & Vitaly Grinkevich

Supplementary information

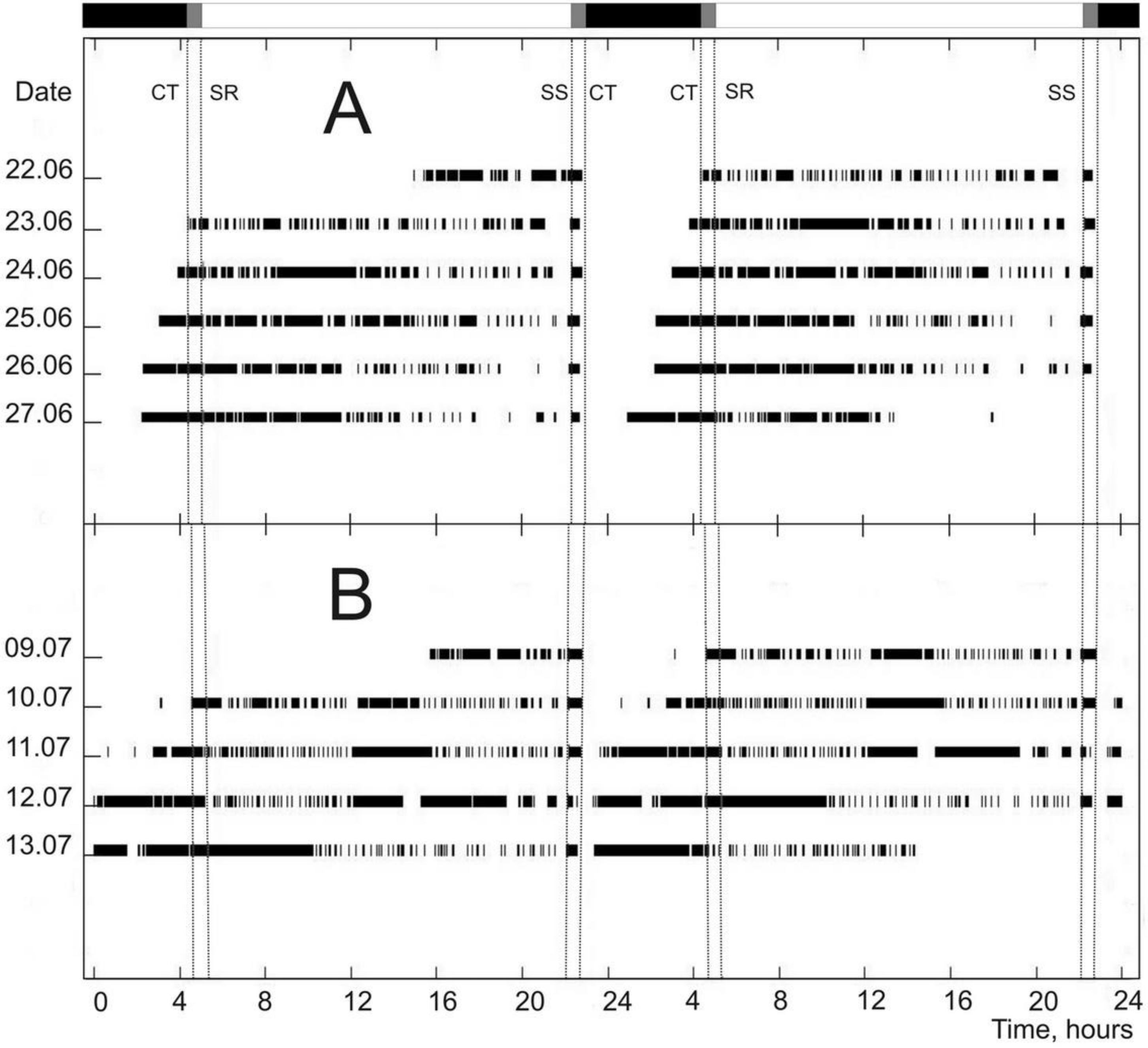
Date
CT
SR
A
SS
CT
CT
SR
SS
22.06
23.06
24.06
25.06
26.06
27.06
B
09.07
10.07
11.07
12.07
13.07
0
4
8
12
16
20
24
4
8
12
16
20
24
Time, hours

Fig.1

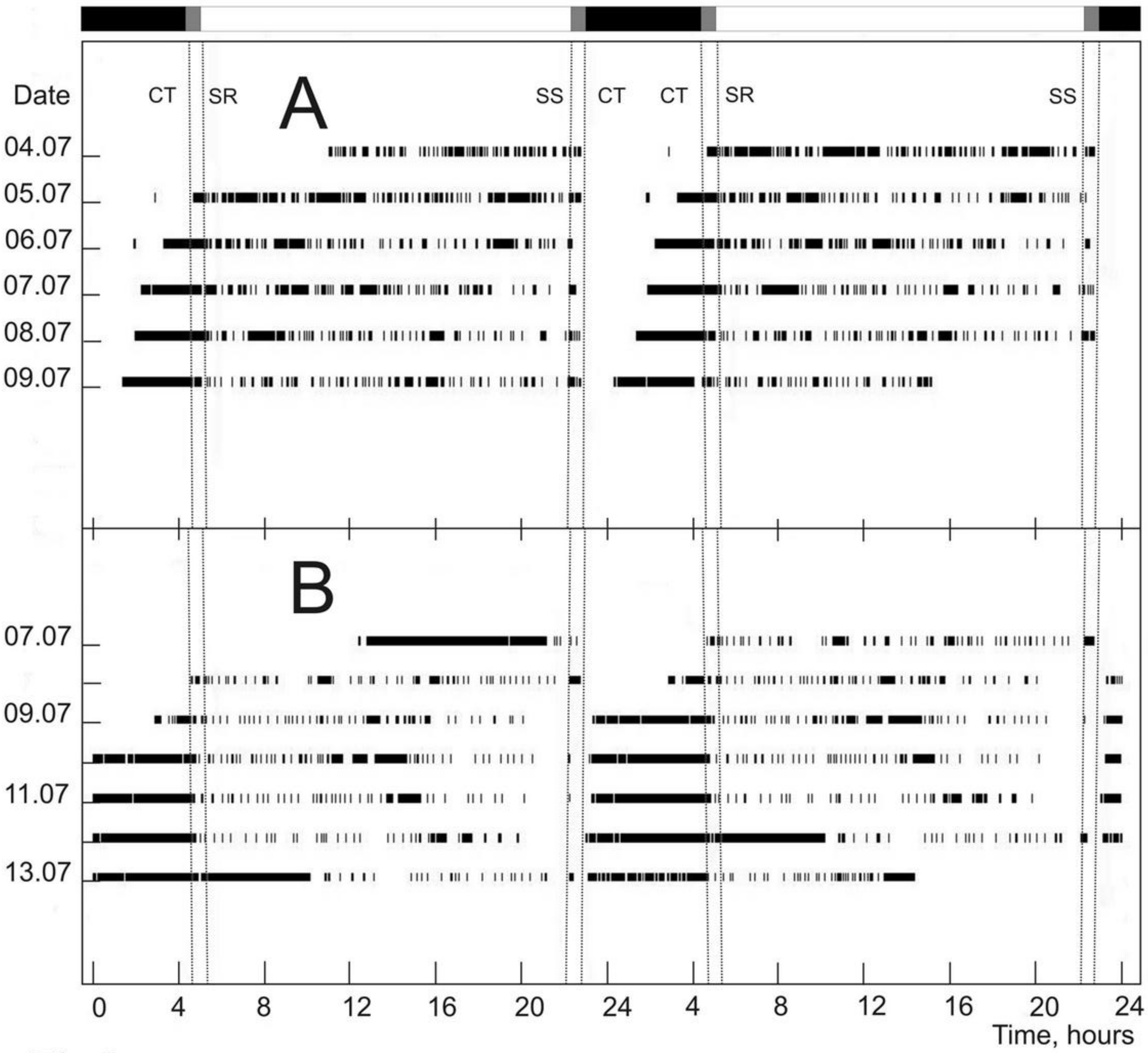
Date
CT
SR
A
SS
CT
CT
SR
SS
04.07
05.07
06.07
07.07
08.07
09.07
B
07.07
09.07
11.07
13.07
0
4
8
12
16
20
24
4
8
12
16
20
24
Time, hours

Fig.2

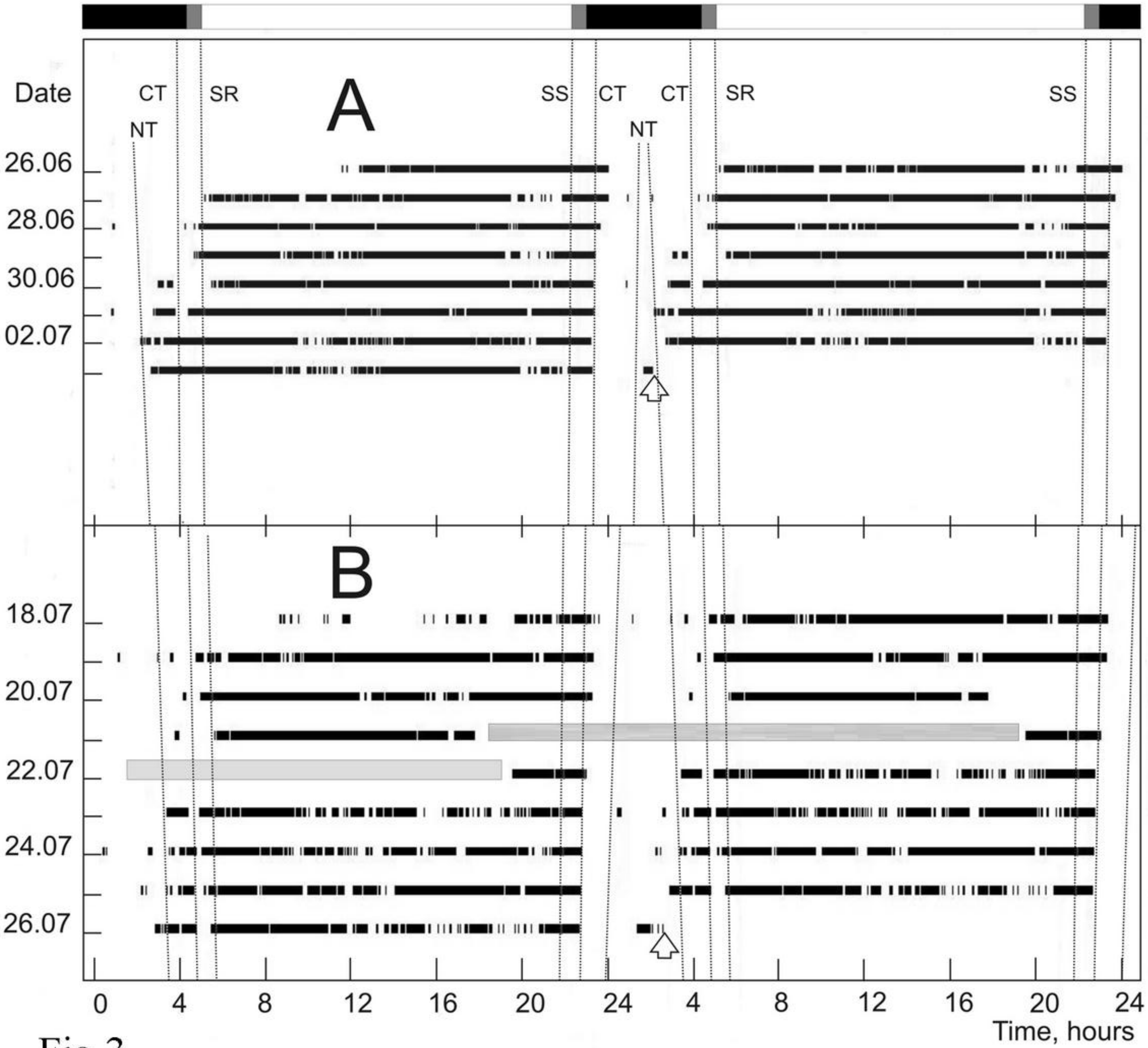

Date
CT
SR
SS
CT
CT
SR
SS
NT
NT
A
26.06
28.06
30.06
02.07
B
18.07
20.07
22.07
24.07
26.07
0
4
8
12
16
20
24
4
8
12
16
20
24
Time, hours

Fig.3

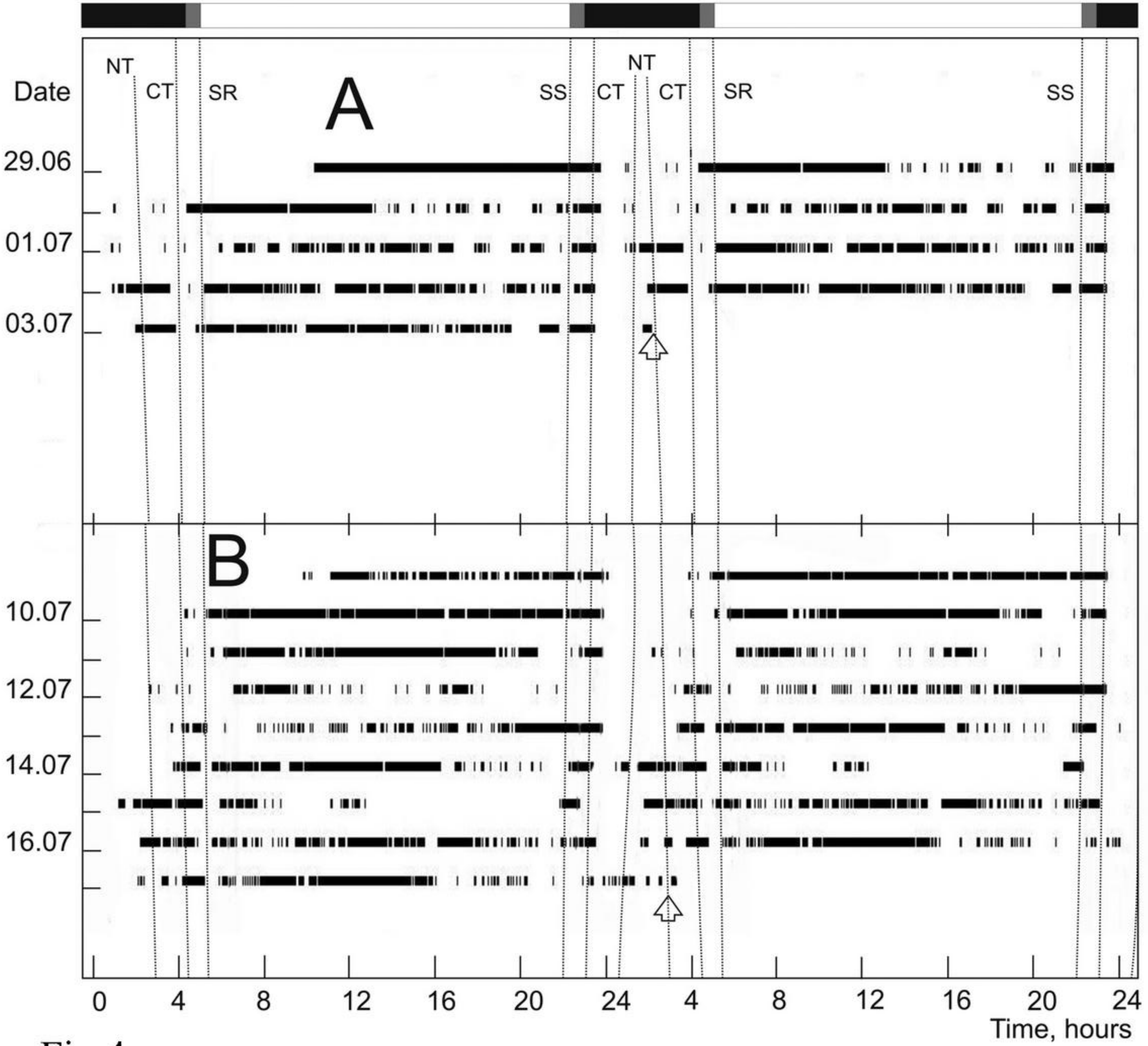
Date
NT
CT
SR
A
SS
CT
NT
CT
SR
SS
29.06
01.07
03.07
B
10.07
12.07
14.07
16.07
0
4
8
12
16
20
24
4
8
12
16
20
24
Time, hours
Fig.4

Interrupted breeding in a songbird migrant triggers development of nocturnal locomotor activity

Andrey Mukhin, Dmitry Kobylkov, Dmitry Kishkinev & Vitaly Grinkevich

Supplementary figure captions

Figure S1. Double plotted actograms of two local birds housed indoor conditions. Upper bar is Light Dark bar. Dotted lines represent the limits: SR – sunrise , SS – sunset, CT – end or beginning of twilight transition. A – bird XY 08697; B – bird XG 01181.

Figure S2. Double plotted actograms of two translocated birds housed indoor conditions. A – bird XN 84672, B – XN 84679. Other symbols are as in Figure S1.

Figure S3. Double plotted actograms of two local birds housed outdoor conditions. CT – limit of civil twilight, NT – nautical twilight. Arrow at last night shows a release moment. Grey bar at B is missing data. A – bird XY 08697, B – XN 84648. Other symbols are as in Figure S1.

Figure S4. Double plotted actograms of two translocated birds housed outdoor conditions. A – bird XG 03149, B – XG 03165. Other symbols are as in Figure S1 and S2.
